# Respiratory Syncytial Virus (RSV) RNA loads in peripheral blood correlates with disease severity in mice

**DOI:** 10.1186/1465-9921-11-125

**Published:** 2010-09-15

**Authors:** Juan Pablo Torres, Ana M Gomez, Shama Khokhar, Vijay G Bhoj, Claudia Tagliabue, Michael L Chang, Peter A Kiener, Paula A Revell, Octavio Ramilo, Asuncion Mejias

**Affiliations:** 1 Department of Pediatrics, Division of Infectious Diseases, University of Texas Southwestern Medical Center, 5323 Harry Hines Blvd, Dallas, TX 75390, USA; 2 Department of Pathology, University of Texas Southwestern Medical Center and Children's Medical Center Dallas, 5323 Harry Hines Blvd, Dallas, TX 75390, USA; 3 Department of Molecular Biology, University of Texas Southwestern Medical Center, 5323 Harry Hines Blvd, Dallas, TX 75390, USA; 4 Medimmune Inc., One Medimmune way, Gaithersburg, MD 20878, USA; 5 Department of Pediatrics, Division of Infectious Diseases and Center for Vaccines and Immunity, The Research Institute at Nationwide Children's Hospital, The Ohio State University, 700 Children's Drive, Columbus, OH 43205, USA

## Abstract

**Background:**

Respiratory Syncytial Virus (RSV) infection is usually restricted to the respiratory epithelium. Few studies have documented the presence of RSV in the systemic circulation, however there is no consistent information whether virus detection in the blood correlates with disease severity.

**Methods:**

Balb/c mice were inoculated with live RSV, heat-inactivated RSV or medium. A subset of RSV-infected mice was treated with anti-RSV antibody 72 h post-inoculation. RSV RNA loads were measured by PCR in peripheral blood from day 1-21 post-inoculation and were correlated with upper and lower respiratory tract viral loads, the systemic cytokine response, lung inflammation and pulmonary function. Immunohistochemical staining was used to define the localization of RSV antigens in the respiratory tract and peripheral blood.

**Results:**

RSV RNA loads were detected in peripheral blood from day 1 to 14 post-inoculation, peaked on day 5 and significantly correlated with nasal and lung RSV loads, airway obstruction, and blood CCL2 and CXCL1 expression. Treatment with anti-RSV antibody reduced blood RSV RNA loads and improved airway obstruction. Immunostaining identified RSV antigens in alveolar macrophages and peripheral blood monocytes.

**Conclusions:**

RSV RNA was detected in peripheral blood upon infection with live RSV, followed a time-course parallel to viral loads assessed in the respiratory tract and was significantly correlated with RSV-induced airway disease.

## Background

RSV is the most common cause of acute lower respiratory tract infection in infants and the leading cause of hospitalization in this age group [1,2]. The clinical manifestations of the disease are thought to be a result of the direct viral cytopathic effect on the respiratory epithelium and the host immune response leading to significant inflammation of the respiratory tract [3-6].

Few reports, mostly in immunocompromised patients, have documented the possible dissemination of RSV outside the respiratory tract [7-12]. However, there are no studies that have examined in detail the potential relation between the presence of RSV RNA in the systemic circulation and whether it is correlated with RSV-induced acute airway disease. Other RNA respiratory viruses, such as the SARS-coronavirus, rhinoviruses, seasonal influenza or avian influenza (H5N1) viruses have been associated with detection of viral RNA in whole blood, blood fractions, serum or plasma in a subset of patients with acute or fatal disease, suggesting that viral dissemination may be associated with poor outcomes [11,13-17].

We used a well-established experimental model of RSV infection to examine the significance of RSV dissemination in disease pathogenesis. We determined 1) whether RSV could be detected in peripheral blood during the acute phase of the disease, 2) its time course of detection compared with the upper and lower respiratory tract, and 3) whether the RSV-induced systemic cytokine response and clinical parameters of disease severity (airway obstruction and lung inflammation) were correlated with blood RSV RNA loads.

## Materials and methods

### Virus, growth conditions and RSV quantification

Human RSV A2 (ATCC-1540) was grown on Hep-2 cells. RSV loads from brochoalveolar lavage (BAL) and nasal wash samples were measured by plaque assay with lower limit of detection of 1.7 log_10 _PFU/mL as described [18,19]. RSV loads in BAL, whole lung, nasal wash and peripheral blood samples were measured by real-time PCR with lower limits of detection of 10 copies/reaction, as described [20,21].

### Animals and Inoculation

Seven-week old BALB/c mice were inoculated with 100 μL 10^7.6-8.3 ^PFU/mL of RSV, or heat inactivated RSV as described [18,19,21]. Uninfected controls were inoculated with 100 μL of 10% EMEM. All experiments were performed following the Institutional Animal Care and Research Advisory Committee guidelines.

### Experimental design and sample collection

Four to six mice per time point, per group were evaluated on days 1, 3, 4, 5, 6, 7, 10, 14 and 21 after inoculation. Mice were inoculated with: 1) live RSV; 2) heat-inactivated (HI)-RSV or 3) EMEM. In a second set of experiments, a group RSV-infected mice was treated with an anti-RSV monoclonal antibody (moAb; motavizumab) administered intraperitoneally (IP; 2.50 mg/per mouse or 100 mg/kg in 100 μl) at 72 h post-inoculation [22]. Mice were euthanized with an IP injection of ketamine and acepromazine prior to cardiac puncture. Blood, nasal wash, BAL and whole lung samples were collected for viral load quantification by culture and real-time PCR. To assess the severity of the pneumonia and the distribution of RSV antigens in the lungs we performed lung histologic evaluation and immunohistochemical (IHC) staining. Systemic CCL2, CXCL1 (innate immunity cytokines), IFN-γ (Th-1) and IL-4 (Th-2) mRNA expression was measured sequentially from days 1 to 7 post-inoculation.

Blood samples (~500 μL/mouse) were obtained by cardiac puncture, mixed in RNA stabilization reagent (Tempus^® ^solution; ABI, Foster City, CA) and stored at -20°C until analysis. On day 5, the peak of RSV disease in this model [18,19,21], peripheral blood samples from two separate experiments were placed in EDTA tubes (BD vacutainer) for RSV antigen detection by IHC staining. Nasal wash and BAL specimens were obtained by infusing 500 μl of 10% EMEM through a 25-gauge needle as previously described [19,23]. Lungs were rinsed with 3 mL of PBS 1× prior to sample collection and fixed with either 10% buffered formalin for histologic evaluation or placed in RNAlater stabilization reagent (Quiagen, Valencia, CA) for viral load quantification [20,21]. Results represent aggregate data from four independent experiments; each included 4-6 mice per time-point per group.

### Pulmonary Function Tests and Lung Histopathology

Enhanced pause (Penh), a measure of airway obstruction (AO), was assessed daily until day 10, and then weekly until day 21 using a whole-body plethysmograph (Buxco Inc, Wilmington, NC) [19,21,24]. For histologic evaluation lung samples were fixed in formalin, embedded in paraffin, and whole-mount sections were stained with H&E. Histopathologic scores (HPS) were determined by a pathologist who was unaware of the infection status of the mice. This scoring system has been previously validated in the RSV mouse model [18,19,21].

### Immunohistochemical Staining (IHC)

Whole lung samples and blood cells were processed following a similar protocol. Briefly, samples were fixed in 10% buffered formalin for 30 min and re-suspended in liquefied histogel. Four-μm sections blocks were cut and placed onto positively charged slides. After deparafinization, sections were incubated at 95-100°C for 36 min for antigen retrieval. After antibody block, a primary goat anti-RSV polyclonal antibody (Biodesign International, Saco, ME) was incubated for 1 h at room temperature followed by incubation with a rabbit secondary antibody (Abcam, Cambridge, MA) and with horseradish peroxidase conjugated anti-rabbit for 24 min (Ventana, Tucson, AZ). Slides were developed using a DAB chromogen-based detection kit from Ventana and hematoxylin used as a counterstain. Lung sections from RSV infected and uninfected mice were used as positive and negative controls respectively.

### Real-time Polymerase Chain Reaction (PCR)

One sample per mouse was evaluated as a single specimen and RNA detection performed by an operator who was blinded to the sample's identification. Samples subjected to RNA detection met the requirements for quantity and quality/purity assessed with the NanoDrop spectrophotometer (Wilmington, DE). No false positives were detected in a total of 1,007 samples analyzed. RNA was obtained from nasal wash, BAL and whole-lung specimens and extracted using ion-exchange mini-columns (Qiagen, Valencia, CA). Whole blood RNA was extracted using the Tempus Spin RNA kit (ABI, Foster City, CA) according to the manufacturer's instructions. RNA reverse transcription, amplification and quantification of a conserved region of the N gene was performed as described [20-22].

### Semi-quantitative real-time PCR for Cytokine analysis

Quantification of blood IFN-γ (Mm 00801778_m1), IL-4 (Mm 00445259_m1), KC/CXCL1 (Mm 00433859_m1), MCP-1/CCL2 (Mm 99999056_m1), was performed by real-time PCR in ABI 7300 HT sequence detector. Relative mRNA expression was calculated using the comparative ΔΔCt method [21,25,26].

### Statistical Methods

Sigma Stat 2008 software (SPSS Science, San Rafael, CA) was used for analyses. Differences between groups were tested using the one-way ANOVA or Kruskal-Wallis test according to data distribution. When these tests demonstrated a significant difference between groups (p < 0.05), the Tukey test to correct for multiple comparisons was used. Pearson or Spearman Rank Order test were used for correlations. In all analyses, a two-tailed p value < 0.05 was considered to be statistically significant.

## Results

### Dynamics of RSV RNA detection in the in the upper and lower respiratory tract

**Figure 1:**
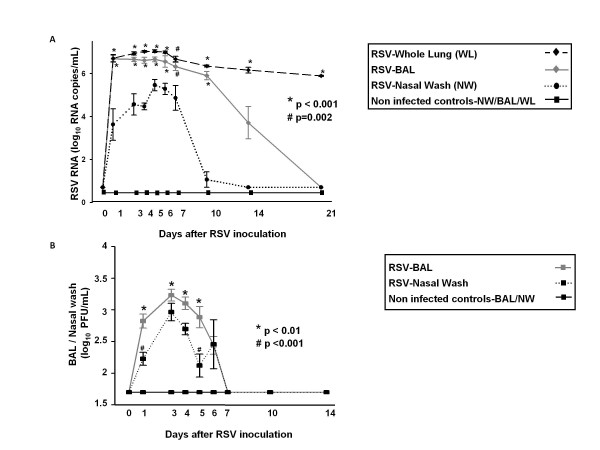
**Dynamics of RSV RNA detection in the upper and lower respiratory tract**. RSV was measured in nasal wash (NW), bronchoalveolar lavage (BAL), whole lung (WL) samples from 4-14 mice per time point per group from 4 independent experiments. Mice were intranasally inoculated with 10^7.6-8.3 ^PFU in 100 μL of RSV or sterile media (non-infected controls). **(A) **Compared with non-infected controls, which had no RSV RNA detected at any time point, RSV RNA loads detected by real-time PCR in WL and BAL were significantly higher than RSV loads detected in NW. Values represent means (± SD) of RSV log_10 _RNA copies/mL. Comparisons were made by One way ANOVA; * p < 0.001. **(B)**. By plaque assay, RSV loads were significantly higher in BAL samples compared with NW on days 1 and 5 post-inoculation. From days 1 to 5 only viral loads measured in BAL samples were higher than those assessed in non-infected controls. Values represent means (± SD) of RSV log_10 _PFU/mL. One way ANOVA was used for comparisons; * p < 0.05.

We first assessed the time course of RSV RNA detection by real-time PCR and the presence of replicative virus by plaque assay in the upper (nasal wash) and lower respiratory tract (BAL and whole lung) of RSV infected mice and sham inoculated controls. In mice infected with live RSV, RSV RNA loads measured in whole lung and BAL samples were comparable and significantly higher than RSV RNA loads measured in nasal wash samples from day 1 to 10 post-inoculation. By day 10, RSV RNA was no longer detected in nasal wash specimens but remained significantly elevated in whole lung samples until day 21 post inoculation (Fig. 1A).

To document the presence of replicative virus, viral loads were measured by plaque assay in BAL and nasal wash samples during the acute infection. Viral loads in both BAL (p = 0.03) and nasal wash samples (p < 0.01) increased from day 1 to days 3-4 post-inoculation, providing evidence of active viral replication, then decreased and remained below the limit of detection of the assay after day 7 post-inoculation, as previously shown in this model [18,19,21]. Overall, viral loads measured in BAL samples were higher than in nasal wash. This difference reached statistical significance on days 1 and 5 post-inoculation (Fig. 1B).

As previously reported, mice inoculated with HI-RSV had significantly lower RSV RNA loads in BAL and whole lung samples compared with mice infected with live RSV and no viable virus was recovered by plaque assay (data not shown) [21].

### RSV RNA is detected in peripheral blood during acute RSV infection and follows a time-course similar to RSV RNA loads measured in the respiratory tract

**Figure 2:**
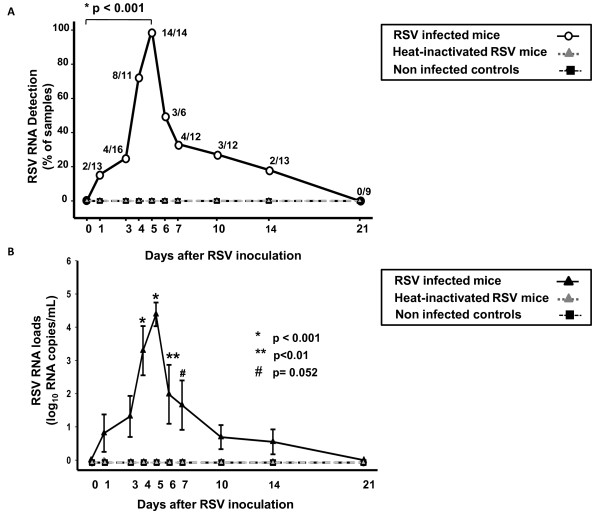
**Time-course detection of RSV RNA in peripheral blood during Acute RSV disease**. RSV RNA was measured in peripheral blood samples sequentially obtained in RSV infected mice, mice inoculated with heat-inactivated RSV and uninfected controls from day 1 to 21 post-inoculation. Mice inoculated with HI-RSV and non-infected controls did not have RSV RNA detected in the blood at any time point evaluated. **Fig. 2. A **The Y-axis represents the percentage of positive samples detected in each experimental condition and the X-axis the time points evaluated. Numbers at each time-point represent the ratio of positive RSV RNA samples/total number of RSV-infected mice evaluated. RSV RNA detected in blood significantly increased from day 1 to day 5 (Z-test, p < 0.001). **Fig. 2. B. **The Y-axis represents the peripheral blood RSV RNA loads in log_10 _copies/mL over time. Blood viral loads in mice infected with live-RSV gradually increased from day 1 to day 5 post-inoculation, the peak of viral detection and viral load quantification (One-way ANOVA p < 0.01).

Once we characterized in detail the dynamics of RSV RNA detection in the upper and lower respiratory tract, we first determined whether RSV RNA could be detected in peripheral blood and second we quantitated the amount of virus present in mice infected with live-RSV, heat-inactivated RSV and sham inoculated controls. From day 1 to 14 post-inoculation RSV RNA was detected in blood samples only in mice infected with live RSV. Detection of peripheral blood RSV RNA significantly increased from 15% of positive samples on day 1, to 72% of samples on day 4, peaked on day 5 with detection in 100% of samples and decreased over time with no blood RSV RNA detected in any RSV-infected mouse beyond day 14 (Fig. 2A). In mice infected with live-RSV blood viral loads gradually increased from 0.8 (± 2.02) log_10 _RSV RNA copies/mL on day 1, to 4.3 log_10 _RSV RNA copies/mL on day 5 (± 1.46) post-inoculation. The highest blood RSV RNA loads were detected on days 4-7, which represent the peak of clinical disease severity in this model (Fig. 2B) [18,19,21]. Mice inoculated with HI-RSV or sham-inoculated controls had no RSV RNA detected in the blood at any time point (n = 110 samples evaluated).

The dynamics of RSV RNA in the blood mirrored RSV loads detected in the respiratory tract, although at significant lower concentrations (p < 0.05). On day 5, the peak of RSV RNA detection in peripheral blood, blood RSV RNA loads significantly correlated with nasal wash and whole lung RSV RNA loads (p < 0.05; r = 0.65-0.76), but not with those measured in BAL specimens.

### RSV Antigens are detected in Peripheral Blood Monocytes

**Figure 3:**
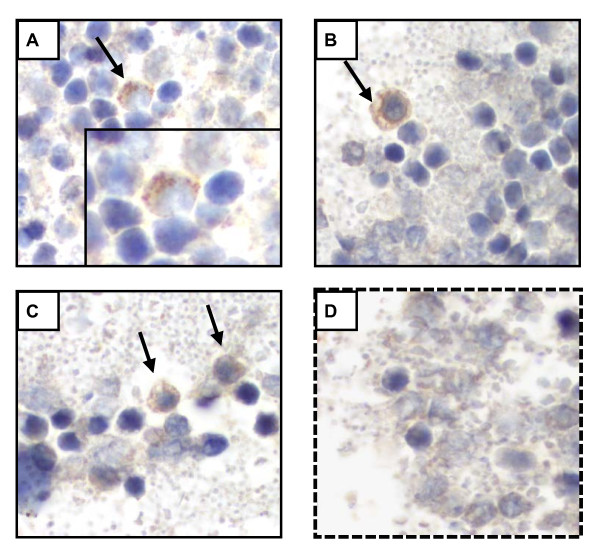
**RSV Antigens are detected in Peripheral Blood Monocytes by Immunohistochemical (IHC) staining**. Peripheral blood was collected in RSV-infected mice (n = 8) and non-infected controls (n = 6) from two independent experiments on day 5 post inoculation. Four-micron sections were IHC stained for RSV with a polyclonal antibody. **Fig. 3A-C **demonstrates peripheral blood monocytes with positive staining for RSV in the cytoplasm. Uninfected mice did not demonstrate any positive staining **(Fig 3D)**.

We attempted to grow RSV from peripheral blood samples on days 4 and 5 post-inoculation, but the standard plaque assays performed did not yield positive results. Alternatively and to confirm the presence of RSV in peripheral blood using a different method we performed immunohistochemical (IHC) staining of blood immune cells obtained from RSV infected mice and non-infected controls. One specimen per mouse (4 mice per group) from two independent experiments was harvested individually on day 5 post-inoculation. Using an anti-RSV polyclonal antibody, IHC staining demonstrated focal and strong positive signal for RSV antigens in the cytoplasm of peripheral blood monocytes in 5 out of 8 RSV infected mice (Fig. 3A-C). The identification of monocytes in peripheral blood was based on their morphology (small cells, with small cytoplasm and small vacuoles) and was performed by an experience pathologist unaware of the infection status of the animals. Cell-blocks from uninfected controls (n = 6) did not demonstrate any positive staining (Fig. 3D). Three different pathologists reviewed the slides in a blind fashion and they all agreed with the results.

### Systemic Cytokine Profiles During Acute RSV Infection

**Figure 4:**
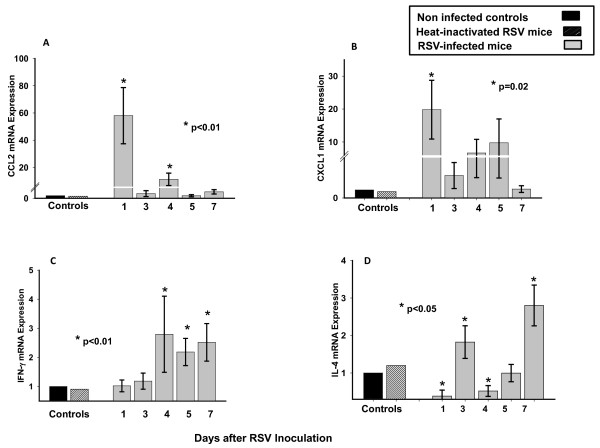
**Dynamics of Cytokine Expression in blood during Acute RSV Infection**. Blood CCL2, CXCL1, IFN-γ and IL-4 mRNA expression was assessed from 4-6 mice per time-point per group in 3 independent experiments from days 1 to 7 after inoculation (**Fig**. **4A-D**). Mice were intranasally inoculated with live-RSV (solid grey bars), heat-inactivated RSV (hatched bars), or 10% EMEM (solid black bars). Values are represented as means ± SD. One-way ANOVA was used for comparisons with non-infected controls mice (*p < .001). When this test demonstrated a significant difference between groups the Tukey test to correct for multiple comparisons was used and considered significant for a p < 0.05.

Once we identified peripheral monocytes bearing RSV antigens, we assessed the systemic expression of CCL2/MCP-1 and CXCL1/KC, which are chemoattractant for monocytes and neutrophils, and IFN-γ and IL-4, as representative examples of Th-1 and Th-2 cytokines. Blood expression of these innate and adaptive immunity cytokines, all implicated in the pathogenesis of acute RSV disease [27-29], were sequentially measured from day 1 to 7 post-inoculation. In addition, we determined whether the systemic expression of these cytokines correlated with RSV RNA loads measured in peripheral blood. Experimental groups included mice inoculated with live RSV, HI-RSV, and non-infected controls (Fig. 4A-D).

Compared with non-infected controls and mice inoculated with HI-RSV, blood CCL2 and CXCL1 mRNA peaked on day 1 (p < 0.01), they leveled off on days 4-5, and decreased by day 7 post-inoculation in mice infected with live RSV (Fig 4A and 4B). On the other hand, the systemic expression of IFN-γ increased on day 4 and remained significantly elevated thereafter (Fig. 4C). IL-4 expression was below the limit of detection or was detected at very low levels from day 1 to 5, but showed a significant increase on day 7 post-inoculation compared with controls. Expression levels of these 4 cytokines were comparable in non-infected controls and mice inoculated with HI-RSV at all time points evaluated (Fig. 4A-D).

There was a significant but inverse correlation between blood RSV RNA loads and blood expression of CCL2 and CXCL1 on day 4 post-inoculation (p < 0.05, r = -0.9/-1).

### Blood RSV RNA Loads Correlates with the Severity of Acute RSV Disease

To determine the significance of blood RSV RNA detection in the context of disease pathogenesis, we assessed disease severity by measuring pulmonary function abnormalities and lung inflammation, and determined whether they were correlated with peripheral blood RSV RNA loads.

**Figure 5:**
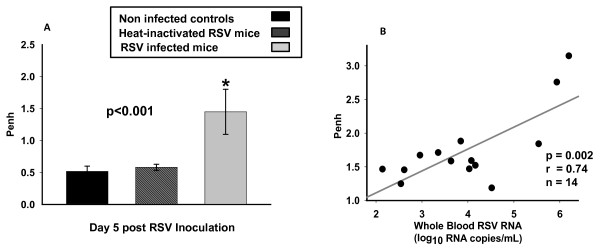
**Blood RSV RNA detection Correlates with Acute Airway Obstruction**. **Fig. 5A**. Airway obstruction assessed by Penh (Y axis) significantly increased in mice infected with live RSV. On day 5 post-infection Penh values were significantly higher in RSV infected mice compared with mice inoculated with HI-RSV and non-infected controls n = 12-16; One way ANOVA (p < 0.001). **Fig. 5B**. Penh values correlated with peripheral blood RSV-RNA loads in RSV infected mice on day 5 post-inoculation (p < 0.01; r = 0.7; n = 14). Correlations were computed using the Pearson's correlation coefficient.

Penh values, a validated measure of airway obstruction [18,19,21], were significantly increased in RSV-infected mice compared with controls and mice inoculated with HI-RSV from day 1 to 14 post-inoculation (p < 0.01) as previously shown [19,21]. On day 5 post-inoculation, Penh values in mice infected with live RSV (Penh; 1.45 ± 0.35) were significantly higher than in mice inoculated with HI-RSV (Penh; 0.58 ± 0.04) and non-infected controls (Penh; 0.51 ± 0.08) (Fig 5.A). Penh values significantly correlated with peripheral blood RSV RNA loads, on day 4 (r = 0.67; p = 0.002), day 5 (r = 0.74; p = 0.002) (Fig. 5.B) and day 10 (r = 0.73; p = 0.005) post-inoculation.

**Figure 6:**
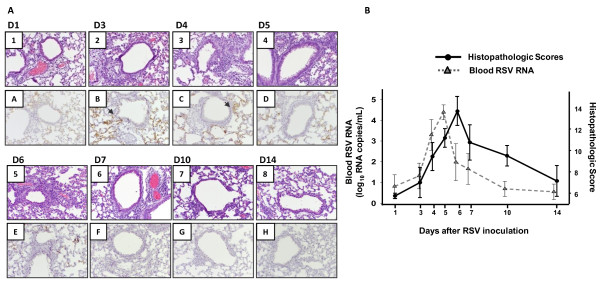
**Lung Histopathology, RSV antigenic distribution and Correlations with blood RSV RNA loads**. **Fig 6A **[1-8]: From days 1 to 7 post-inoculation, changes included perivascular edema and margination of neutrophils (day 1), which progressed to a sparse perivascular infiltrate and later to dense perivascular and peribronchial/peribronchiolar inflammatory infiltrates composed of lymphocytes, macrophages and scattered neutrophils. At its peak (days 5 to 7), these inflammatory infiltrates extended into surrounding alveolar septa in a stellate manner with patchy involvement of the parenchyma and abundant macrophages, occasional lymphocytes and neutrophils in alveolar spaces. On days 10 and 14 the persistent changes included inflammatory infiltrates located around vessels and airways, consisting of lymphocytes and macrophages, but no neutrophils or eosinophils. **Fig 6A. [A-H]**. By immunohistochemical staining (IHC) the alveolar epithelium showed a diffuse faint linear staining on day 1, was stronger and widespread on days 3-4, and decreased by days 6-7. In contrast, alveolar macrophages' IHC was negative on day 1, occasionally positive on day 3, strong and diffusely stained on days 4 to 6 and decreased by day 7. Both the alveolar epithelium and alveolar macrophages were negative for RSV IHC on days 10 and 14 post-infection. **Fig 6B**. Histopathologic scores significantly increased in mice infected with live-RSV from day 1 (HPS mean ± SD; 5.7 ± 0.5) to day 5 (HPS 11.2 ± 2.1; p = 0.005), day 6 (HPS = 13.6 ± 2.3; p = 0.001) and day 7 (HPS = 10.7 ± 3.3; p = 0.02). There was no correlation between the peak of blood RSV RNA loads and lung inflammation.

As we previously shown in this model, the severity of airway obstruction gradually and significantly increased from day 1 to day 5, the peak of airway disease [18-22]. Lung inflammation was assessed sequentially on days 1, 3, 4, 5, 6, 7, 10 and 14 post-infection. Compared with sham-inoculated controls (Histopathologic Scores, HPS = 0-1), mice inoculated with live RSV showed significantly greater HPS, with a dense inflammatory infiltrate and severe pneumonia, which peaked on days 5-7 post-inoculation (p < 0.01) (Fig. 6A; 1-8). To define the distribution of RSV antigens in the lungs, IHC staining was performed using a polyclonal RSV antibody. Immunostaining showed an increasingly positive stain for RSV in the alveolar epithelium from day 1 to days 3-4 and decreased by days 6-7 post-inoculation. On the other hand, IHC in alveolar macrophages was negative on day 1, occasionally positive on day 3, strong and diffusely positive on days 4 to 6 and also decreased by day 7. Both the alveolar epithelium and alveolar macrophages were negative for RSV staining on days 10 and 14 post-infection (Fig 6A; 6A-H). In contrast to airway obstruction, there was no significant correlation between the severity of lung inflammation (HPS) and blood RSV RNA loads on day 5, illustrating the different time course of these two parameters of disease severity (Fig. 6.B).

### Treatment with anti-RSV neutralizing antibody reduced blood RSV RNA loads and improved acute airway obstruction

**Figure 7:**
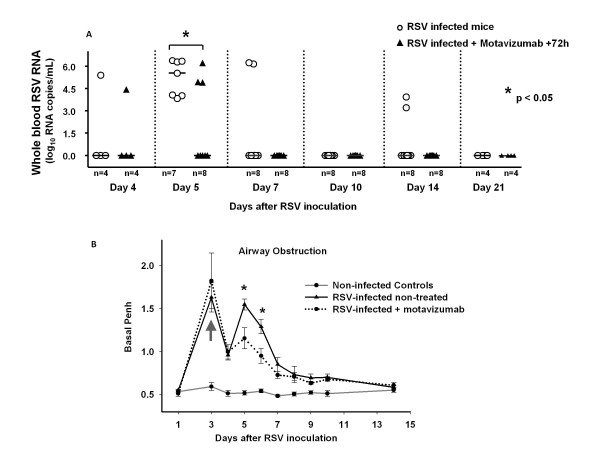
**Administration of anti-RSV moAb reduces the length and peak of RSV RNA detection in the blood and is associated with improved airway obstruction**. **Fig. 7A. **Blood RSV RNA loads were measured by real-time PCR on day 4, one day after treatment with anti-RSV moAb, and then on days 5, 7, 10, 14 and 21 in mice infected with RSV (white circle), mice infected with RSV and treated 72 h later with anti-RSV moAb (black triangle) and controls (not represented). Data shown are the result of two separate experiments each with 4 mice per time-point per group. Comparisons were made by Mann-Whitney Rank Test *p < 0.05. **Fig. 7B. **Non-infected controls (grey circle), RSV-infected mice + treated 72 h later with anti-RSV moAb (black circle) and RSV infected-non treated mice (black triangle) were evaluated for AO, by measuring Penh via whole-body plethysmography. Penh was significantly lower in mice inoculated with RSV and treated with anti-RSV moAb at days 5 and 6 post-inoculation compared with RSV infected non-treated mice. Comparisons made by One way Analysis of Variance * p < 0.05. Red arrow indicates the time of therapeutic intervention with anti-RSV moAb at 72 h post RSV inoculation.

To further assess the significance of blood RSV RNA detection in disease pathogenesis, we determined whether treatment with an anti-RSV moAb had an effect on RSV RNA loads in peripheral blood. Treatment with anti-RSV moAb at 72 hours post-inoculation reduced both the duration and peak of blood RSV RNA loads. Mice treated with the moAb had blood RSV RNA loads detected only from day 4 to 7, and showed significantly lower peak blood RSV RNA loads than non-treated RSV-infected mice on day 5. After day 6, RSV RNA was no longer detected in the blood of any mouse treated with anti-RSV moAb (Fig. 7.A). In addition compared with untreated RSV-infected mice, airway obstruction was significantly reduced from day 4 (24 hours after treatment) to day 7 post-inoculation in RSV-infected mice treated with the moAb. This difference reached statistical significance on days 5 and 6 post-inoculation (Fig 7.B).

## Discussion

Our current understanding of the ability of RSV to spread from the respiratory tract to the systemic compartment, the time course of detection in peripheral blood, and whether this has implications for disease pathogenesis is limited. Using a mouse model of RSV infection, we found that: 1) inoculation with live RSV was consistently associated with detection of RSV RNA in peripheral blood; 2) the time course of RSV RNA detection in peripheral blood mirrored viral replication in the respiratory tract; 3) RSV antigens were detected in peripheral blood monocytes and lung alveolar macrophages during the acute phase of the disease; 4) blood RSV RNA loads correlated with acute airway obstruction; and 5) administration of an anti-RSV antibody significantly decreased the peak and duration of peripheral blood RSV RNA loads and was associated with improved airway obstruction.

Despite that the systemic spread of other paramyxoviruses has been frequently reported in the literature [30,31], limited efforts have been dedicated to characterize the significance of the detection of RSV in peripheral blood in experimental models or patients with naturally acquired infection. Three studies documented the presence of RSV RNA by real-time PCR in blood or peripheral blood mononuclear cells (PBMCs) in a subset of neonates and children with RSV lower respiratory tract infection [8-10]. In those studies, RSV RNA was also found in the blood of a small percentage of children with no evidence of RSV infection, and the association between the detection of RSV in the blood and parameters of disease severity was inconclusive. On the other hand, the detection of RSV RNA in the serum of a 2-month old with RSV bronchiolitis was associated with the development of hepatitis, indicating the possible dissemination of RSV in severe forms of the disease [12]. More recently, a study conducted in hematopoietic cell transplant (HCT) recipients with respiratory virus-associated pneumonia showed that compared with bronchoalveolar lavage (BAL) viral loads, serum RSV, hMPV and influenza virus RNA loads were significantly associated with the need for mechanical ventilation and death, [11]. In the present study, the detection of peripheral blood RSV RNA gradually increased from day 1 to day 5, with detection in 100% of samples on day 5 post-inoculation which correlated with the peak of clinical disease in this model defined by increased airway obstruction.

The detection of RSV antigens by immunofluorescence prior to the implementation of PCR assays has been previously described in PBMCs of children and calves with acute RSV infection [32,33]. In agreement with those studies, using IHC staining we identified peripheral blood monocytes bearing RSV antigens on day 5, the peak of RSV RNA detection in blood. Similarly, on days 4 to 6 post-infection the distribution of RSV antigens in the lungs was predominant in alveolar macrophages. Hence, it is possible that during the acute infection immune cells bearing RSV antigens may migrate from the respiratory tract to the systemic compartment, and this migration may explain the detection of RSV RNA in peripheral blood.

Despite that RSV antigens were detected in peripheral monocytes, blood RSV RNA loads were inversely correlated with blood CXCL1 (neutrophil chemoattractant) and CCL2 (monocyte chemoattractant) on day 4 post-inoculation and there were no correlations with IFN-γ and IL-4, key Th1/Th2 adaptive immunity cytokines [19,29,34]. The fact that CXCL1 and CCL2 peaked early on the infection when blood RSV RNA was detected at very low levels, and inversely correlated with the peak of RSV RNA loads on day 4 may reflect the different dynamics of viral replication and cytokine production in RSV disease.

Unexpectedly, the peak of blood RSV RNA loads did not correlate with the severity of lung inflammation, suggesting that the different components of RSV-induced lung disease may have different pathogenic mechanisms or follow different dynamics. It also suggests that the detection of RSV RNA in the blood might be an earlier event in disease pathogenesis that precedes the establishment of severe pulmonary inflammation. If this is the case, detection of RSV RNA in peripheral blood may help identifying the more severe forms of RSV disease in an early stage.

Our study has a number of limitations. We did not document viral replication in the blood using traditional cell culture, which may be due to the limited sensitivity of the culture assay with small sample volumes. Nevertheless, RSV RNA was consistently detected only in peripheral blood of mice inoculated with live RSV, suggesting that live viral infection is required for pathogenic viral dissemination. We further documented the presence of RSV by a completely different methodology, and identified a cell subtype, monocytes, bearing RSV antigens in peripheral blood. It is possible that the high RSV inoculum used caused severe lung inflammation and cell damage with subsequent leakage of RSV particles, which may have been engulfed by peripheral blood monocytes. However, the decrease of blood RSV RNA loads after treatment directed against RSV suggests that the detection of RSV RNA in peripheral blood truly reflected the spread of the virus to the systemic compartment. Lastly, the identification of RSV RNA was limited to the respiratory tract and peripheral blood but not to other organs, which could have help to indirectly assess the systemic spread of RSV during the acute infection, and warrants further studies to determine whether other organs are also affected in severe RSV disease.

## Competing interests

OR has served as a consultant and has received research grants from Merck, Medimmune and Abbott. PAK was employee of Medimmune.

## Authors' contributions

JPT: carried out and lead the experiments, performed data analysis and drafted the manuscript. AMG: Carried out the immunohistochemical studies and gave insightful advice to carry out the experiments. SK: carried out PCR and culture assays. VGB, CT and MLC: performed mouse experiments, pulmonary function tests studies and tissue culture. PAR and PAK: assistance in study design data interpretation. OR participated in the design of the study, data interpretation and helped to draft the manuscript. AM: conceived the study, and participated in its design and coordination, helped with data analysis and interpretation and helped to draft the manuscript. All authors have read and approved the final manuscript.
